# The WAGR syndrome gene PRRG4 is a functional homologue of the *commissureless* axon guidance gene

**DOI:** 10.1371/journal.pgen.1006865

**Published:** 2017-08-31

**Authors:** Elizabeth D. Justice, Sarah J. Barnum, Thomas Kidd

**Affiliations:** Department of Biology/ms 314, University of Nevada, Reno, Nevada, United States of America; New York University, UNITED STATES

## Abstract

WAGR syndrome is characterized by Wilm’s tumor, aniridia, genitourinary abnormalities and intellectual disabilities. WAGR is caused by a chromosomal deletion that includes the PAX6, WT1 and PRRG4 genes. PRRG4 is proposed to contribute to the autistic symptoms of WAGR syndrome, but the molecular function of PRRG4 genes remains unknown. The *Drosophila commissureless* (*comm*) gene encodes a short transmembrane protein characterized by PY motifs, features that are shared by the PRRG4 protein. Comm intercepts the Robo axon guidance receptor in the ER/Golgi and targets Robo for degradation, allowing commissural axons to cross the CNS midline. Expression of human Robo1 in the fly CNS increases midline crossing and this was enhanced by co-expression of PRRG4, but not CYYR, Shisa or the yeast Rcr genes. In cell culture experiments, PRRG4 could re-localize hRobo1 from the cell surface, suggesting that PRRG4 is a functional homologue of Comm. Comm is required for axon guidance and synapse formation in the fly, so PRRG4 could contribute to the autistic symptoms of WAGR by disturbing either of these processes in the developing human brain.

## Author summary

Mutants for the fruit fly *commmissureless* gene (*comm*) dramatically lack connections between the left and right hand sides of the nervous system. This is due to a failure to prevent Robo receptors from reaching the cell surface, where they guide growing axons away from the CNS midline. Comm proteins are not thought to exist outside of insects. By carefully comparing proteins from other species, candidate homologues from vertebrates and yeast were identified. The candidates were tested by expression in the fly nervous system and one gene, PRRG4, was found to affect the phenotype caused by expression of the human Robo1 gene. When Robo genes are expressed in cell culture, they localize to the surface of the cell. PRRG4 was found to be able to re-localize Robo away from the cell surface, a property shared with Comm protein, indicating that they are functional homologues. Human patients with WAGR syndrome often display autistic features and these have been attributed to loss of one copy of PRRG4. Our findings suggest that PRRG4 guides growing axons and that brain wiring patterns may be subtly altered in WAGR patients.

## Introduction

The Commissureless protein (Comm) in *Drosophila* regulates the cell surface expression of Roundabout (Robo) axon guidance receptors by targeting Robos for degradation during secretion through the ER/Golgi network [1], reviewed in [2]. Failure to down-regulate Robo leads to a dramatic phenotype in which axon crossing of the CNS midline is abolished [3]. Conversely, overexpression of *comm* induces ectopic midline crossing through increased removal of Robos [4–6]. Comm is also required for the correct formation of the *Drosophila* brain commissure [7]. Comm is a relatively short protein with a single transmembrane domain and L/PPxY motifs [1, 8]. Comm binds the WW domain containing ubiquitin ligase Nedd4 via L/PPxY motifs [9], but this function appears only to be required for endocytosis activities at the neuromuscular junction [10, 11]. Despite the conservation of the Robo/Slit pathway, homologues of Comm have not been found outside of insects and alternative molecules and mechanisms have been proposed for Robo regulation in the vertebrate spinal cord [12–15].

The vertebrate proline rich and Gla domain genes PRRG1-4, also known as PRGP1, PRGP2, TMG3 and TMG4 respectively [16, 17], encode short transmembrane proteins. PRRG4 protein has been found in the Golgi apparatus and at the cell surface [18–20]. All PRRG proteins contain a Gla domain in which glutamic acid (Glu) residues are γ-carboxylated in the endoplasmic reticulum by γ-glutamyl carboxylase (GGCX) [21, 22] to form γ-carboxyglutamate (Gla) residues. Gla domains coordinate calcium ions to allow binding to membrane phospholipids [23]. Although γ-carboxylation plays a major role in blood clotting, the enzymes required for this post-translational modification are also found in invertebrates, which lack the vertebrate blood clotting cascade, suggesting additional functions [24, 25]. PRRG proteins are expressed highly in tissues such as the spinal cord and so are believed to play roles outside the coagulation cascade [16, 17]. The cytoplasmic domains of PRRG proteins are characterized by PPxY and LPxY motifs that are best known as acting as ligands for WW domain containing proteins [26, 27]. The PRRG proteins are therefore members of a family of transmembrane proteins that can recruit additional proteins or vesicles to the membrane via the Gla domain or L/PPxY motifs.

WAGR (Wilm’s tumor, Aniridia, Genitourinary malformations and mental Retardation) syndrome is a rare genetic disorder caused by haploinsufficiency of the 11p13 chromosomal region [28–30]. The WAGR critical region includes the WT1 and PAX6 transcription factors, which are responsible for the Wilm’s tumor and aniridia phenotypes respectively [31, 32]. WAGR syndrome is frequently accompanied by developmental delay and autism like features. The genes that could contribute to these symptoms include PAX6, SLC1A2, DCDC1 and PRRG4 [33, 34]. In a survey of 31 WAGR patients with autism, all were deleted for PRRG4, a correlation that suggested that PRRG4 is involved in autistic symptoms [33]. The critical region for severe developmental delays and autistic behaviors was subsequently narrowed down to 1.6Mb that includes PRRG4, but not SLC1A2 or DCDC1 [35]. Understanding the function of PRRG4 is therefore a key step in determining whether PRRG4 contributes to the autistic behaviors.

**Fig 1:**
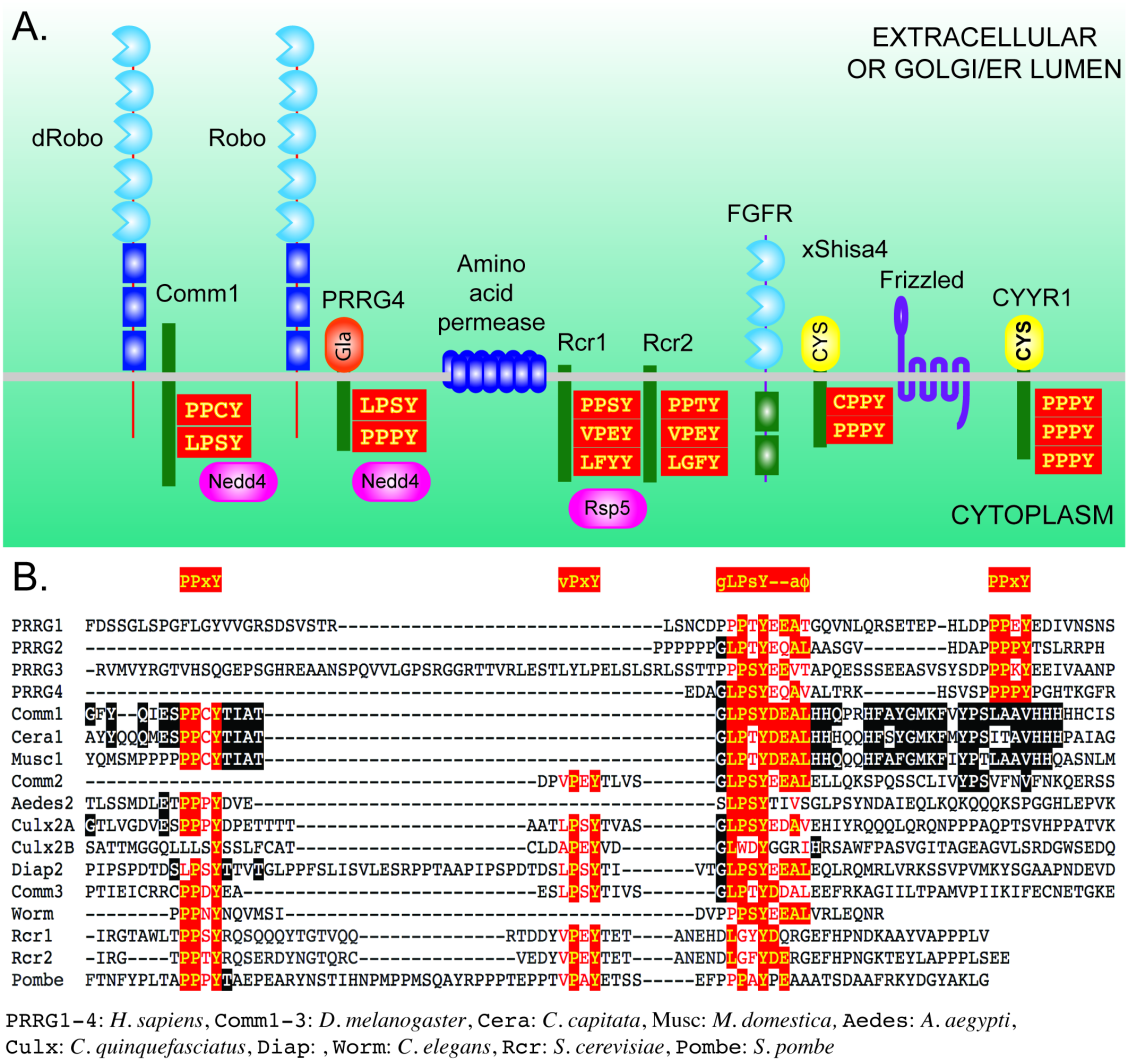
Structures, binding partners and PY motifs of Comm, Rcr, PRRG and Shisa proteins. **A**. Schematic representations of potential Comm homologues and their interacting partners. *Drosophila* Comm1 physically associates with Robo receptors [1], but it is not known if this interaction is direct or mediated by additional proteins. Comm1 regulation of fly Robo1 requires the transmembrane domain of Robo1 [62]. An interaction between PRRG4 and Robo receptors is predicted by the work in this study. The PY motifs of Comm1 interact with the WW domain of the Nedd4 E3 ubiquitin-protein ligase [9]. The PY motifs of the human PRRG4 protein also interact with the WW domain of Nedd4 [18]. The interaction with Nedd4 is only implicated in endocytosis of Robo from the cell surface and not in regulation of Robo exocytosis [10]. In yeast the Rcr1 and Rcr2 proteins regulate the cell surface expression of amino acid permease [38], and Rcr1 physically interacts with the Rsp5 ubiquitin ligase via cytoplasmic PY motifs [37]. Shisa proteins physically interact with Fibroblast Growth Factor Receptors (FGFR) and Frizzled Wnt receptors and prevent their trafficking to the cell surface [41]. Shisa proteins are distinguished by Cys-rich N-terminal domains, a feature shared by CYYR1 [40]. **B**. Amino acid alignments of the proline rich (PY) motifs in the cytoplasmic domains of candidate Comm homologues. These motifs are generally of the form PPxY or LPxY, where P is proline, Y is tyrosine and L is leucine and x is any amino acid. Comm1 has an extended PY motif, GLPSYDEAL, that is critical for Comm1 function. The core LPxY motif together with conserved acidic and hydrophobic residues is shared with the PRRG proteins. In PRRG proteins, a PPxY motif occurs after this extended motif, whereas in invertebrate Comm proteins, additional PY motifs occur before the LPSY sequence. Outside of the motifs, conservation is remarkably low even in other insect species. The species used for the alignment are shown underneath.

During literature searches for short transmembrane proteins containing L/PPxY motifs, we noticed similarities between Comm, the Rcr1 and Rcr2 genes in yeast, and the PRRG, CYYR and Shisa families in vertebrates (Fig 1A). We tested representatives of these families for the ability to affect axon guidance in the fly ventral nerve cord. We find that expression of PRRG4 in a sensitized background induces midline crossing. When expressed in COS cells, PRRG4 reduces the surface localization of Robo proteins. Our results place PRRG4 in an evolutionarily conserved gene family that regulate the cellular localization of cell surface proteins.

## Results

### Identification of putative Comm homologues from other species

The GLPSYDEAL motif of Comm has been shown to be essential for Comm function in midline crossing [1], and constitutes an extended version of an L/PPxY motif [36](Fig 1B). We searched the literature for PY motif proteins from other species and compared their structure to that of Comm. In *S*. *cerevisiae*, the Rcr1 and Rcr2 proteins contain PPSY and VPEY motifs and have an overall structure resembling that of Comm. The VPEY motif binds the Rsp5 ubiquitin ligase with PPSY having a cooperative function [37]. This activity is likely required for endocytotic trafficking of yeast membrane proteins [38]. In *Drosophila*, the Nedd4 ubiquitin ligase binds Comm by either the LPSY or PPCY motifs, but with an *in vivo* preference for LPSY [9]. The Nedd4 interaction is required for endocytosis at the neuromuscular junction formation [11], but not the regulation of Robo during midline crossing [10]. In an interesting parallel, Rsp5 is not required for activity of Rcr1 in chitin deposition. These similarities led us to test Rcr1 and Rcr2 for activity in the fly nervous system.

Yeast has been used to screen for human genes regulating plasma membrane protein trafficking and CYYR1 gene was identified in this manner [39]. CYYR1 is characterized by a cysteine (Cys) rich N-terminal, three conserved Cys residues within the transmembrane domain as seen for Comm2 and other insect proteins, and three PPxY motifs (Fig 1A). CYYR1 appears to be a member of the large Shisa-like protein family (STMC6), all of which are short single pass transmembrane proteins involved in protein trafficking and degradation [40]. Shisa proteins physically interact with Frizzled and FGF receptors in the ER/Golgi, preventing their maturation and trafficking to the cell surface in *Xenopus* and mice [41, 42]. Disruption of these developmentally important pathways could potentially mask subsequent effects on axon guidance. However, Comm proteins lack Cys residues in their extracellular domain so are less likely to be homologues. We tested two divergent members, Xenopus Shisa4 and human CYYR1 to check for the ability to regulate Robo. After testing these genes, we observed that the uncharacterized gene CG15760 is likely the *Drosophila* homologue of Shisa-like gene family, based on the C*C*CC*CC arrangement of Cys amino acids in the putative extracellular/lumenal domain (S1 Fig) [40].

Searching for other PY motif proteins, our attention was drawn to the PRRG proteins, two of which lack signal sequences like Comm. All have PPxY and LPxY motifs in their cytoplasmic domains, with PRRG4 having an exceptional match to the critical Comm GLPSYDEAL motif: GLPSYEQAV, when conservative substitutions for the negatively charged and hydrophobic amino acids are taken into account (Fig 1B). The human PRRG2 LPxY sequence closely matches that of Comm homologues from the housefly and the Mediterranean fruit fly. The PPxY motif comes after the LPxY motif in these genes, and an SH3 binding motif is also present in PRRG2 and PRRG4 [17, 18]. Finally, we noticed an uncharacterized *C*. *elegans* gene C17G10.7 with two PPxY motifs, one with acidic residues following the tyrosine (S2 Fig). However, an alternative alignment for the predicted C17G10.7 protein has four putative transmembrane domains so may align with LAPTM4 proteins instead (S2 Fig) [39]. Nevertheless, the predicted protein had additional homologies at the N- and C- termini that led to it being included in testing.

### Bioinformatic evidence for a putative Gla domain in commissureless

**Fig 2:**
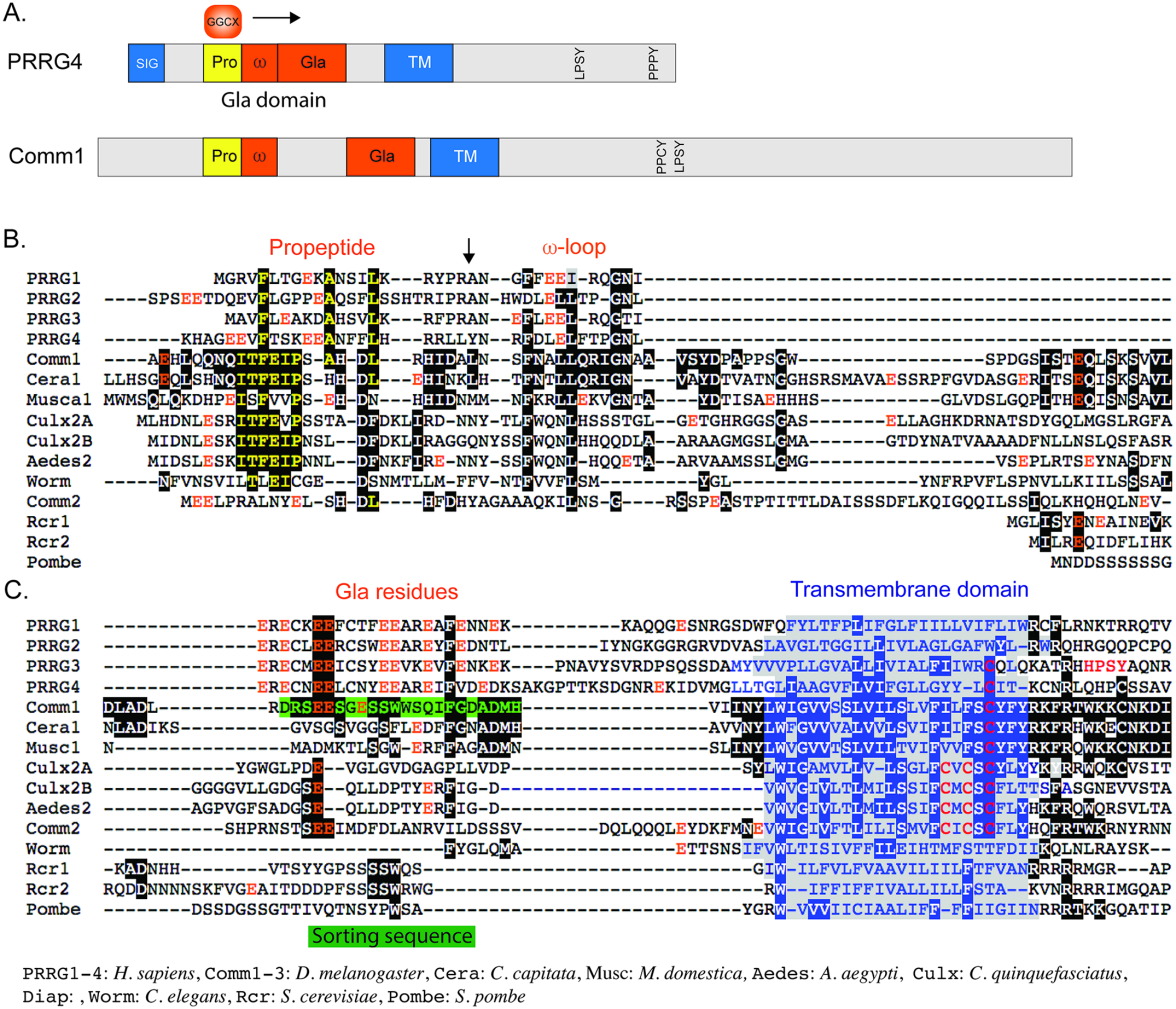
Comparison of the gamma-carboxylation domains of PRRG proteins with Comm family members. **A**. Schematic comparing the domain structures of Comm1 and PRRG4. PRRG4 has a signal sequence (SIG) that is lacking in Comm1 (and also PRRG1 and PRRG3). The enzyme γ-glutamyl carboxylase (GGCX) binds to the propeptide (PRO) and proceeds to modify glutamic acid residues in a processive fashion, frequently starting in the adjacent or ωloop or keel domain (ω) before proceeding to the conserved Gla domain (Gla) that contains multiple glutamic acid residues. In our alignment of PRRG4 and Comm1, the ω and Gla domains are separated in Comm1. The transmembrane domains (TM) and PY motifs are also indicated. **B**. Amino acid alignment of the extracellular/lumenal domains of PRRG and Comm proteins. The critical residues in propeptides are phenylalanine (F), alanine (A) and leucine (L) at positions -16, -10 and -6 relative to the propeptide proteolytic cleavage site (vertical arrow). These residues are conserved in the *Drosophila* Comm1 protein, and appears partially conserved in other insect Comm proteins. The ω-loop contains glutamic acid (E) residues flanked by conserved phenylalanine and leucine residues, and the latter are conserved throughout Comm proteins. **C**. Gla domains are characterized by a high frequency of glutamic acid residues, and conserved Cys and phenylalanine (Phe) residues. Comm1 displays weak homology to the Gla domain as a phenylalanine and a pair of glutamic acid residues are in conserved positions. In Comm1, the region that aligns with the Gla domain corresponds to the juxtamembrane lumenal peptide identified by Keleman et al. as critical to Comm1 localization and function [10]. The phenylalanine and a glutamic acid residue are present in some but not all insect Comm proteins. The yeast Rcr proteins lack Gla domains but display some extremely limited homology to Comm1. A conserved Cys is present near the cytoplasmic end of the transmembrane domain in all Comm proteins and in the PRRG3 and PRRG4, both of which can regulate Robo in cell culture (albeit partially in the case of PRRG3). The species used to supply the sequences for the alignment are shown at the bottom.

As noted in the introduction, PRRG proteins contain an N-terminal Gla domain consisting of Glu residues that are γ-carboxylated by GGCX. The GGCX and VKOR enzymes required for γ-carboxylation are present and functional in flies, but surprisingly GGCX knockouts have no apparent phenotypic defects [43–45]. Gla domains contain a propeptide sequence bound by GGCX, a hydrophobic region called the “keel” or ω-loop that binds phospholipids giving Gla domains membrane binding properties [46], and a highly conserved region of Glu and Cys residues that coordinate calcium ions. The activity of GGCX on its substrates is greatly enhanced by the presence of a propeptide sequence that is proteolytically removed after GGCX has moved along the protein [47, 48]. The propeptide consensus consists of a highly conserved phenyalanine residue at -16, an alanine at -10 and a leucine at position -6 relative to the proteolytic cleavage site, as well as additional conserved hydrophobic amino acids [49, 50]. An N-terminal motif, ITFEIP, conserved among Comm proteins is centered on a Phe residue and is followed by Ala and Leu residues only slight offset from the vertebrate consensus, suggesting this region could function as a propeptide (Fig 2B). GGCX functions in a processive manner and usually begins modifying Glu residues immediately downstream of the propeptide, which frequently occur within the keel or ω-loop. The sequence FLEEL in PRRG3 represents this initial substrate and is identical to a sequence frequently used to measure GGCX activity and the influence of the propeptide [51]. Comm proteins show distant homology to this initial substrate, although the Glu residues are missing (Fig 2B). The keel region may insert directly into the membrane being bound by the Gla domain, so the hydrophobic residues are likely the most important [52, 53]. Deletion of this region of Comm greatly reduces Comm function *in vivo* indicating its importance [54]. The remainder of the Gla domain coordinates calcium ions via the Gla residues. In Comm, a short sequence adjacent to the transmembrane domain is essential to Comm activity (labeled the “sorting sequence” in Fig 2B) [10]. As before there is weak homology to the Gla domain (Fig 2C), with the proposed ω-loop and the rest of the domain physically separated in Comm. Given the distant homologies to Gla domains in Comm, as well as the conservation of the LPxY motif, we tested PRRG1-4 genes in the fly nervous system.

### Expression of candidate Comm homologues in the *Drosophila* ventral nerve cord

**Fig 3:**
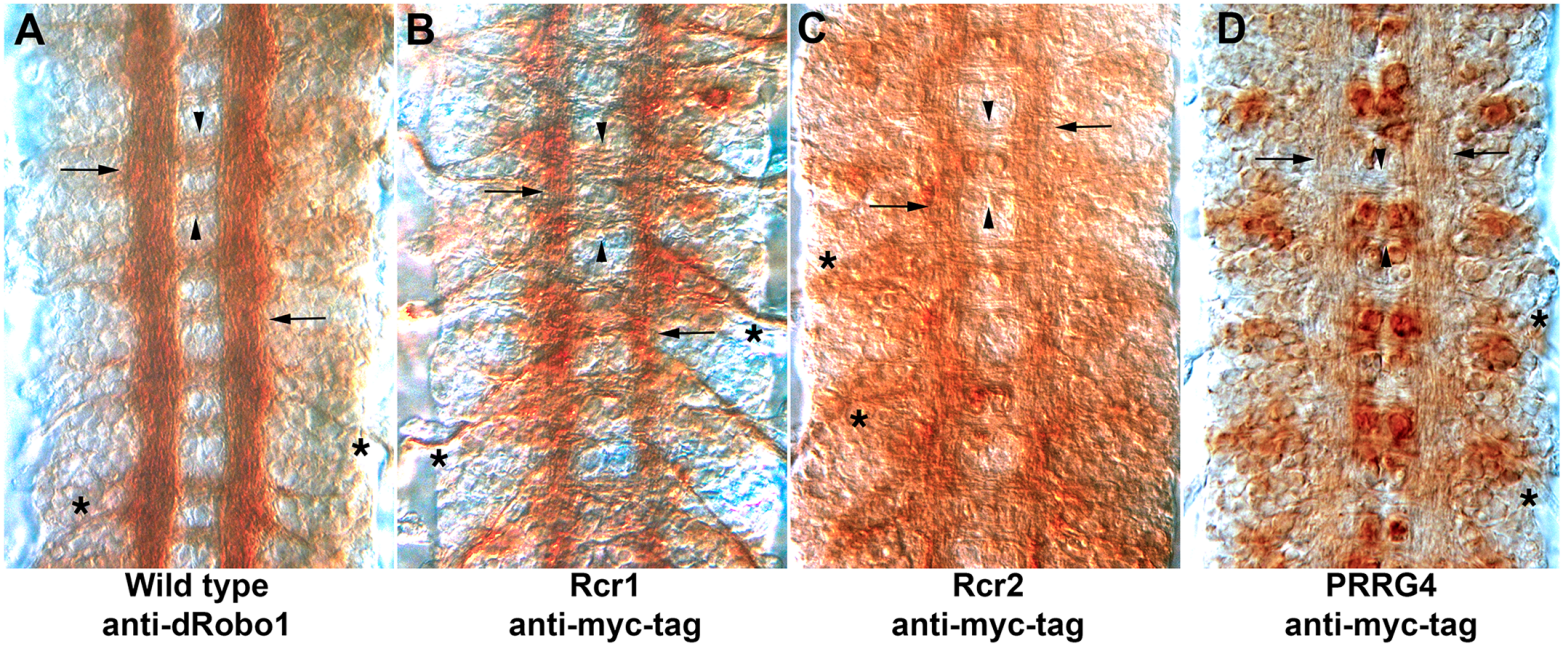
Axonal localization of yeast Rcr proteins. Dissected *Drosophila* embryonic nerve cords imaged with Nomarksi or differential interference (DIC) microscopy that allows unstained axons to be visualized. The CNS axon scaffold forms a characteristic ladder like pattern and lies on top of the cell bodies that make up the nerve cord. For mis-expression experiments, a single copy of the *sca-GAL4* pan-neural driver was combined with a single copy of the indicated UAS transgene **(B-D)**. **A**. dRobo1protein is primarily localized to the longitudinal axon tracts (arrows) with much less staining in the commissures that cross the CNS midline (arrowheads). The axons of the motor nerve roots are also labeled (asterisks). **B**. Expression of epitope tagged yeast Rcr1 in CNS axons. There is some staining in the underlying cell bodies, but expression clearly is stronger in the longitudinal axon tracts (arrows) compared to the commissures (arrowheads). The motor nerve roots display strong axonal localization (asterisks). **C**. Very light axonal staining can be seen for yeast Rcr2 with much stronger expression in the underlying cell bodies (arrows, arrowheads). The nerve roots have faint staining (asterisks). **D**. PRRG4 protein expression is restricted to cell bodies and is absent from axons. Trace amounts of PRRG4 protein may be present in the axons but the primary reason the axons are visible is due to DIC microscopy. Cell body expression is most evident underneath the commissures (arrowheads), but also lateral to the longitudinal tracts (arrows). The nerve roots lack detectable PRRG4 expression (asterisks).

The open reading frames of the selected genes (*S*. *cerevisiae* Rcr1, Rcr2, *C*. *elegans* C17G10.7, *X*. *laevis* Shisa4, and *Mus musculus* CYYR1 and PRRG1-4) were synthesized with a *Drosophila* codon bias. All open reading frames had a myc epitope tag added at the carboxy-terminus, were subcloned into the pUAST expression vector and used to generate transgenic fly lines. The lines were tested by pan-neural expression using the *scabrous-GAL4* (*sca-GAL4*) driver and staining for the myc epitope to confirm expression. Comm protein is found in cell bodies, cytoplasmic vesicles and axons [8], and we expected that a candidate homologue might show the same pattern. As yeast lacks a nervous system, we did not expect to see axonal localization of Rcr1 or Rcr2. However, we found that by stage 16 of embryonic development yeast Rcr1 and to a lesser extent, Rcr2, localized to longitudinal axons in a manner reminiscent of Robo1 protein (Fig 3B and 3C). This raises the possibility that the Rcr proteins may be weakly interacting with Robo proteins. In contrast, the vertebrate PRRG4 protein remained in the neuronal cell bodies (Fig 3D). As trafficking and cell surface localization of Gla domain proteins can be dependent on γ-carboxylation [55, 56], it is possible that the fly GGCX enzyme does not properly process PRRG4.

### PRRG4 expression increases midline crossing

**Fig 4:**
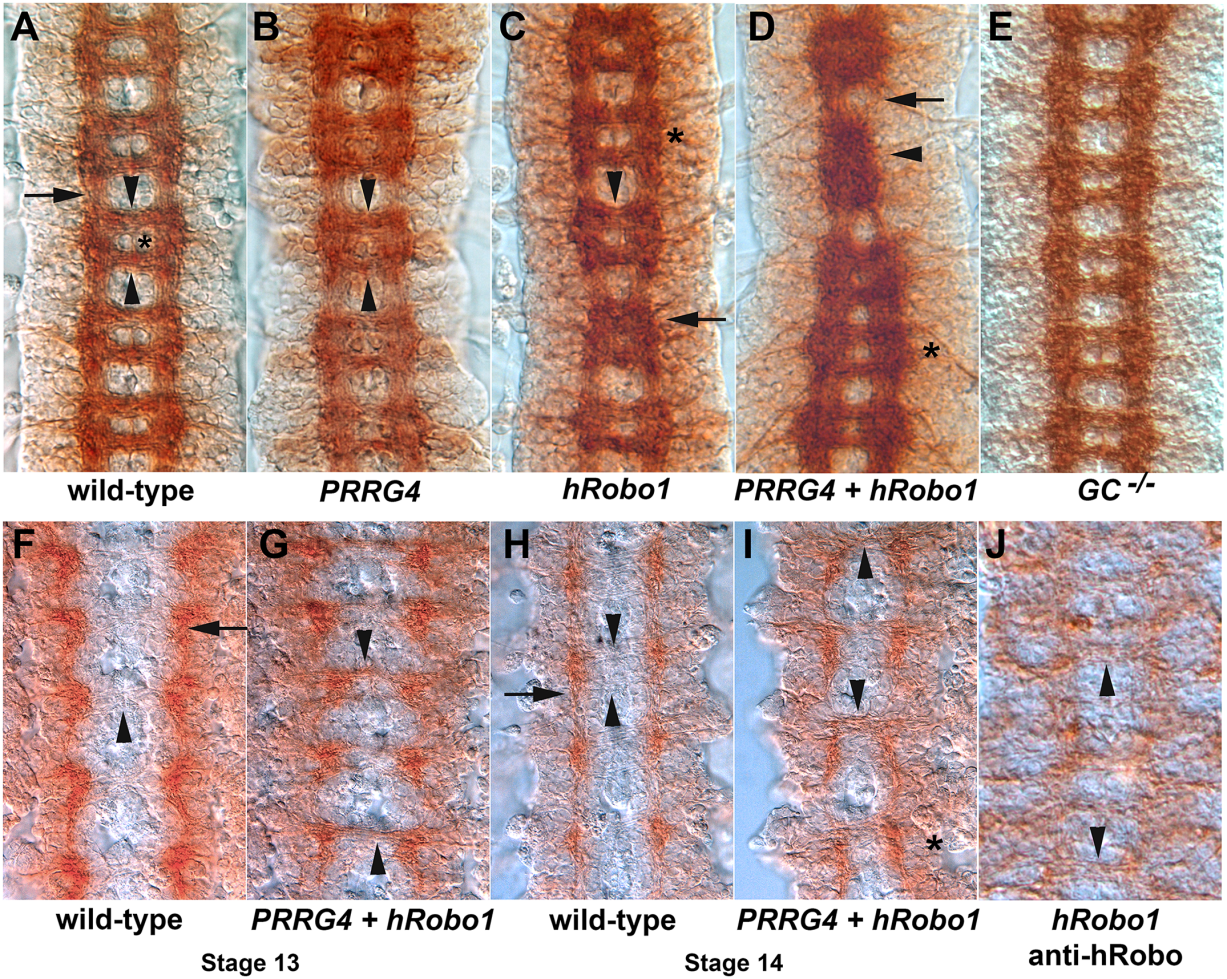
PRRG4 induces axon guidance errors when co-expressed with human Robo1 in the *Drosophila* ventral nerve cord. Dissected *Drosophila* embryonic ventral nerve cords stained with the monoclonal antibodies BP102, which stains the CNS axon scaffold (**A-E**), or anti-dRobo1 13C9 (**F-I**), or anti-vertebrate Robo1 (**J**). Both stains are brown. For over-expression experiments, a single copy of the *sca-GAL4* pan-neural driver and the indicated UAS transgene is present **(B-D, G, I)**. **A**. The wild type axon scaffold of a stage 16 embryo exhibits a regular arrangement of commissures that cross the CNS midline (arrowheads) and longitudinal tracts that project along the anterior-posterior body axis (arrow). The anterior and posterior commissures in each segment are separated by the cell bodies of midline glia and motor neurons (asterisk). **B**. Pan-neuronal expression of PRRG4 results in a failure to fully separate the commissures leading to a fuzzy appearance of commissures (arrowheads). **C**. Pan-neuronal expression of human Robo1 causes the axon scaffold to partially collapse on the midline in some segments (arrow). In other segments the commissures appear thicker (arrowhead) and in several segments the width of the axon scaffold is reduced even though the commissures remain separated (asterisk). **D**. Co-expression of PRRG4 and hRobo1 results in strong axon phenotypes, including collapse of axons onto the midline in a manner resembling *slit* mutants (arrowhead), fuzzy and unseparated commissures with disrupted longitudinals (arrow) or fuzzy and only partially separated commissures (asterisk). **E**. Stage 16 embryo homozygous for a null mutation in the γ-glutamyl carboxylase gene (GC). No defects in the axon scaffold were observed. **F**. Stage 13 wild type embryo stained for fly Robo1 protein. The longitudinal portion of the axon scaffold stains brown (arrow), but the commissures lack Robo1 protein (arrowhead). The commissures are just beginning to be separated by the migration of midline glia. **G**. Pan-neural expression of PRRG4 and hRobo1 results in Robo1 protein entering the commissures (arrowheads), a phenotype seen when *comm* is over-expressed. **H**. Stage 14 wild type embryo in which the commissures are separated but lack visible Robo1 staining (arrowhead). The longitudinal tracts have Robo1 staining (arrow). **I**. An embryo expressing both PRRG4 and hRobo1 displays Robo1 protein in the commissures (arrowheads). The phenotypic effects of expressing both PRRG4 and hRobo1 can be witnessed in the asymmetry of the staining and lateral reduction in the width of the scaffold (asterisk). **J**. An embryo with pan-neuron expression of *UAS-hRobo1* using the *scratch-GAL4* driver was stained with anti-Robo1 (Abcam ab7279). Human Robo1 protein is visible in commissural axons (arrowheads), indicating that hRobo1 is not subject to regulation by fly Comm. The data is summarized in Table 1 and the underlying data are shown in S1 Data.

Pan-neuronal over-expression of *comm* in the fly CNS induces ectopic midline crossing that phenocopies *robo* mutants because Robo proteins are downregulated by excess Comm [4–6]. In our hands, CNS axon guidance phenotypes require multiple copies of the *sca-GAL4* driver and the *UAS-comm* transgene. We screened several independent UAS transgene insertions for each candidate Comm homologue by crossing to *sca-GAL4*, recovering the F1 generation and examining the embryos laid. This allowed us to rapidly generate large numbers of embryos potentially carrying more than one copy of the *sca-GAL4* driver and/or the UAS transgene. Staining of the CNS axon scaffold revealed no mis-expression phenotypes for the *CYYR1*, *xShisa4*, *C17G10*.*7*, *Rcr1*, *PRRG1*, *PRRG2* and *PRRG3* genes. *Rcr2* expression resulted in very minor aberrations in the axon scaffold in a very low percentage of embryos. *PRRG4* expression had very rare and subtle phenotypes (Fig 4B), but still stood out from the other transgenes for having a noticeable effect. Very rarely stronger effects ranging from increased midline crossing in single segments to missing commissures were observed. The latter phenotype suggests that PRRG4 might act as a dominant negative. We repeated the *PRRG4* experiments with the *scratch-GAL4* promoter, which has a similar expression pattern as *sca-GAL4*, but may express for longer, but saw no increase in phenotypes. The low frequency of *PRRG4* phenotypes suggested that two copies of the GAL4 and UAS transgenes are required to obtain phenotypes.

Increasing expression of the *PRRG4* transgene beyond two copies would have been challenging, so we sought out alternative approaches to increase the phenotypic penetrance of *PRRG4* expression. We were concerned that interactions between Robo and Comm might be species specific, as Comm has no effect on zebrafish Robo1 or Robo3 localization in S2 cells [57]. Expressing human *Robo1* (*hRobo1*) in the ventral nerve cord subtly increases midline crossing (Fig 4C, Table 1). This is in contrast to fly *robo1* over-expression, which leads to a commissureless phenotype [58]. Fly and vertebrate Robo proteins can dimerize via their cytoplasmic and extracellular domains [6, 59, 60], and also form heterodimeric complexes with other receptors bridged by Slit [61]. This suggests that hRobo1 may be acting as a dominant negative, interfering with the function of endogenous Robos perhaps by creating inactive heterodimers. Co-expression of PRRG4 with hRobo1 strongly enhanced the midline crossing phenotype (Fig 4D, Table 1, S1 Data). The interaction of the γ-carboxylated PRRG4 protein and hRobo1 suggested that γ–carboxylation might be important for fly nervous system formation. We examined the nerve cords of mutants for the γ-glutamyl carboxylase (GC) gene, but found no defects in the axon scaffold (Fig 4E). In these embryos co-expressing PRRG4 and hRobo1, fly Robo1 protein can be found in the commissures (Fig 4F and 4H). A similar mislocalization is seen in *comm* gain of function embryos [4]. We examined the protein localization of hRobo1 when expressed in the fly ventral nerve cord and found it present in the commissures suggesting it is not regulated by fly Comm (Fig 4J). Of the candidate genes tested, PRRG4 was the strongest candidate for a Robo regulator identified in these tests.

**Table 1:** Quantification of axon guidance defects in PRRG4 overexpression experiments.

Percent of segments demonstrating phenotypes						
**BP102 Phenotype**	*Wild type*	*<robo*	*robo*	*>robo*	*slit*	*Total*
***wild-type***	99	1	0	0	0	100
***sca***::***hRobo1***	48	41	9	2	0	100
***sca***::***hRobo1***, ***PRRG4***	7**	34	42***	16*	1	100

Stage 16 embryonic nerve cords were stained with BP102 and examined for the indicated genotypes. *PRRG4* refers to an *UAS-PRRG4* construct, *hRobo1* to *UAS-human-Robo1* and *sca* to the *scabrous-GAL4* pan-neuronal driver. The embryonic CNS commissures are separated by two cell bodies visible with Nomarski optics per segment. The axon scaffold was scored for wild type morphology, thickened commissures or only one midline cell visible. “*<robo*”, stereotypical *robo1* phenotypes in which the cell bodies separating the commissures are not visible or a minimal gap between the commissures “*robo*”, embryo segments in which the lateral constriction in width was greater than that of *robo* mutants “*>robo*” and segments in which the axons had collapsed onto the midline “*slit*”. See Fig 4 for examples. Total refers to the total number of segments scored for each genotype. Expression of *PRRG4* by *sca-GAL4* alone is predicted not to lead to any phenotypes, as multiple copies of these transgenes only produce a low frequency of phenotypes; enhancing the penetrance and expressivity of *PRRG4* expression was the impetus for co-expression with *hRobo1*. The data for all three genotypes was analyzed with a Kruskal-Wallis one-way ANOVA and all three genotypes were found to be statistically different in all categories (p < 0.0004) except “*slit”*. The data for *sca*::*hRobo1* and *sca*::*hRobo1*, *PRRG4* were directly compared with a Mann-Whitney U test and the “*wild type*”, “*robo*” and “*>robo*” categories were statistically different (p = 0.001, 0.0001 and 0.014 respectively and indicated with asterisk in the table; * p < 0.05, ** p < 0.01, *** p < 0.001).

The underlying data are shown in S1 Data.

### Colocalization of Comm, PRRG and Robo proteins in cell culture

**Fig 5:**
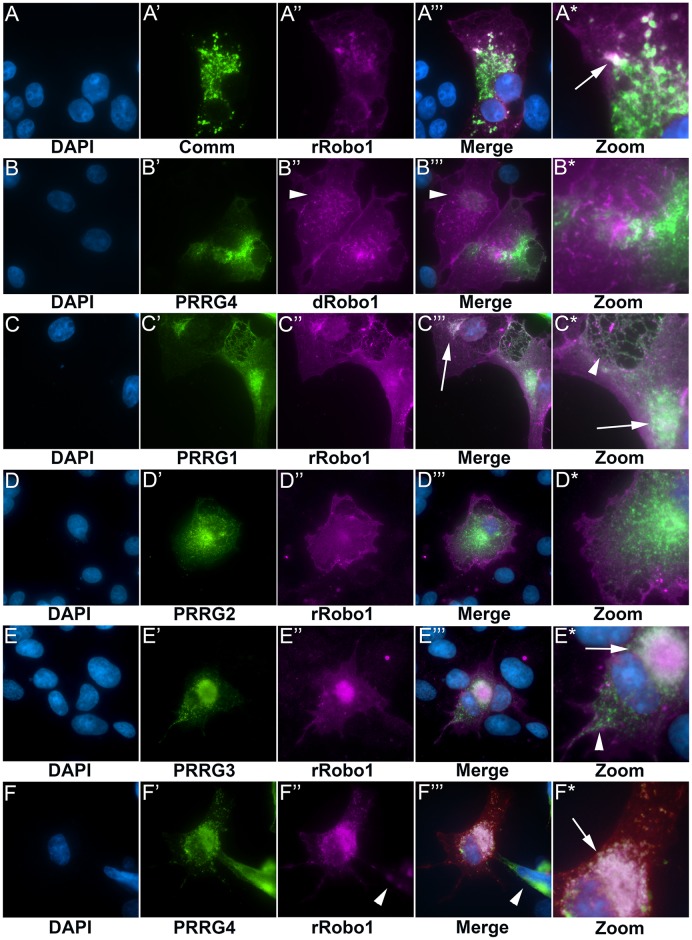
Co-localization of Comm, PRRG and Robo proteins. COS cells were transfected with epitope tagged constructs as indicated under the figure panels. Protein expression was detected by antibody labeling and fluorescence microscopy. Nuclei were detected by DAPI (blue) staining. **A**. Cells transfected with both Comm and rRobo1. Despite the presence of significant amounts of Comm protein (green), rRobo1 remains localized at the cell surface and throughout the cell (magenta), suggesting that these proteins do not interact in this assay. There is a very limited degree of overlap of Comm and rRobo1 expression within the cell (white; arrow in A*) but also a significant lack of overlap in most other areas. **B**. Co-expression of both PRRG4 and dRobo1. In this image, two cells are transfected with dRobo1 (magenta), but only one cell expresses high levels of PRRG4 (green). Comparison of the two cells reveals that the pattern of dRobo1 in the cell with a low level of PRRG4 (arrowhead) shows little difference with the cell expressing PRRG4 suggesting the two proteins do not interact. Almost no overlap (white) is seen in the magnified panel (B*). **C**. PRRG1 and rRobo1 display a slight degree of co-localization in the presumed ER/Golgi when co-expressed (white areas, arrows in C”‘ and C*). In areas not adjacent to the nucleus, strong separation of the two proteins is seen (arrowhead in C*) suggesting they are not interacting. **D**. Co-expression of PRRG2 and rRobo1 leads to little or no co-localization of the proteins. **E**. Expression of PRRG3 can lead to a reduction of rRobo1 on the cell surface (E”) and limited co-localization around the nucleus (arrow in E*). Nevertheless, the two proteins do not co-localize in many parts of the cell (arrowhead in E*). These results suggested that PRRG3 may have a limited capacity to interact. **F**. Co-expression of PRRG4 and rRobo1 results in a strong reduction of cell surface rRobo1 (F”) and co-localization of the two proteins throughout the cell, particularly in the presumed ER/Golgi adjacent to the nucleus (arrow in F*). An adjacent cell expresses a high level of PRRG4 and a low level of rRobo1 (arrowheads in F” and F”‘) suggesting that PRRG4 may be resulting in degradation of rRobo1. These results strongly suggest that PRRG4 and rRobo1 interact in cell culture.

To further investigate the potential PRRG4-Robo interaction, we co-expressed constructs in COS cells and looked for co-localization. We began by testing Comm and rat Robo1 (rRobo1). Robo proteins localize to the cell surface, whereas Comm is primarily in the ER/Golgi (Fig 5A) [1, 8]. Some co-localization occurs but may be because both proteins are in the secretory pathway. The clearance of dRobo1 from the cell surface of COS cells has been used as an assay for Comm function [1, 62], but we saw no evidence that rRobo1 is cleared from the cell surface by Comm suggesting that these two proteins do not interact. Similarly, co-expression of PRRG4 and fly Robo1 (dRobo1) showed limited co-localization and no re-localization of dRobo1 from the cell surface (Fig 5B). Taken together with previous results showing no interaction between Comm and zebrafish Robo1 and Robo3 in S2 cells [57] and our results showing no localization of hRobo1 in the fly ventral nerve cord (Fig 4J), this suggests that interactions between Robo and Comm/PRRG genes have co-evolved since insects and vertebrates split.

We tested all four mouse PRRG proteins for co-localization with rRobo1. PRRG1 and PRRG2 showed minimal or no co-localization with rRobo1, and rRobo1 did not appear to re-localize from the cell surface (Fig 5C and 5D), suggesting these proteins do not interact. We obtained mixed results with PRRG3 as we saw partial co-localization with rRobo1, but also clear separation of staining (Fig 5E). rRobo1 also appeared to be partially cleared from the cell surface (Fig 5E”), and these results may be interpreted as a weak interaction between PRRG3 and rRobo1. We have included additional examples of co-localization to document this effect (S3 Fig). PRRG4 showed a strong co-localization with rRobo1, clearing rRobo1 from the cell surface and co-localizing in the presumed ER/Golgi adjacent to the cell nucleus (Fig 5F). This result strongly resembles that of Comm and dRobo1, suggesting that PRGR4 and rRobo1 interact in cell culture.

### PRRG4 regulates rRobo1 *in vitro*

**Fig 6:**
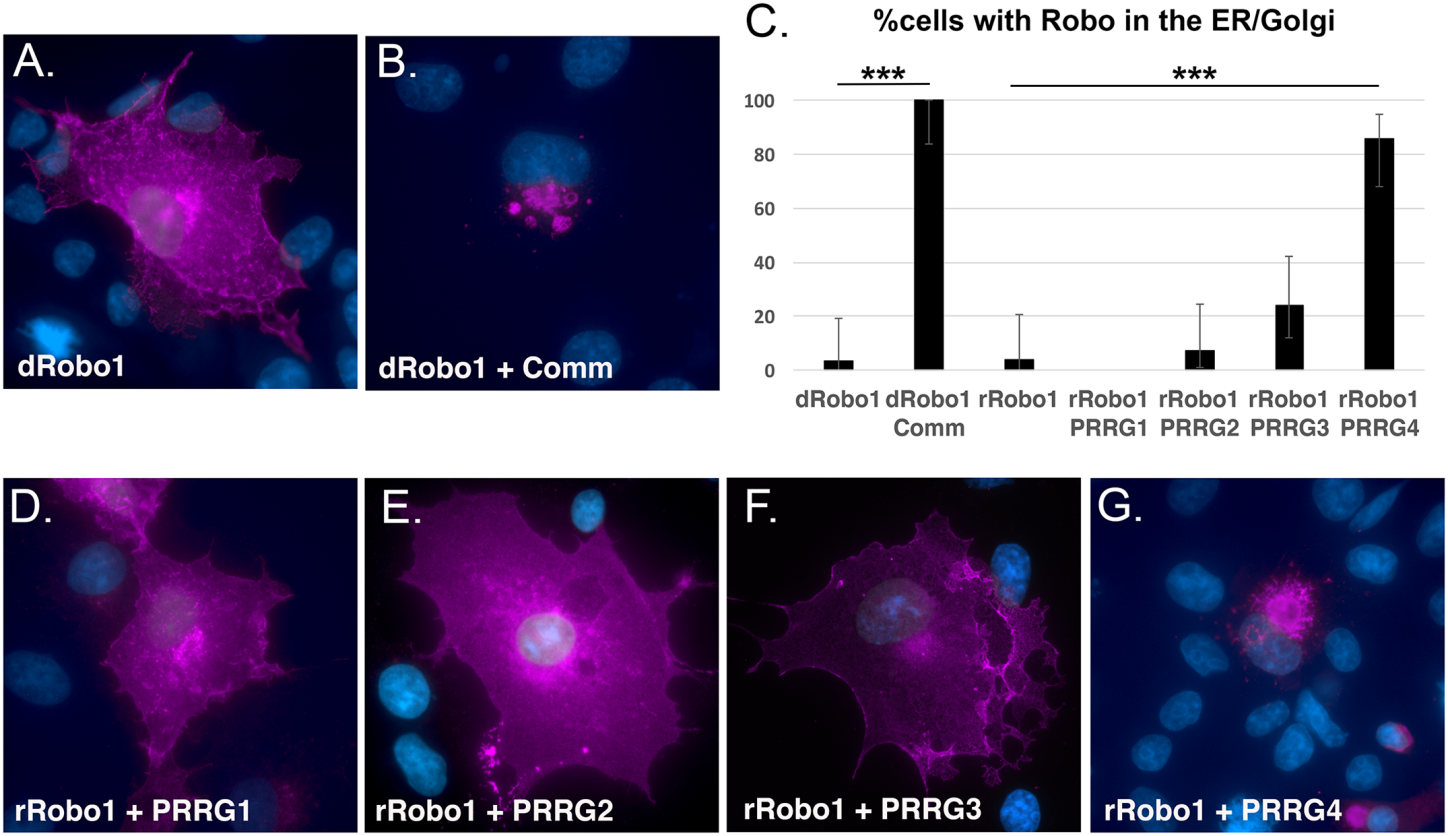
PRRG4 re-localizes rRobo1 from the plasma membrane. COS cells were transfected with plasmids encoding the genes indicated in the panels and antibody stained with anti-Robo in magenta. Cells were counterstained with DAPI (blue) to reveal the nucleus. **A**. *Drosophila* Robo1 (dRobo1) and **B**. Co-expression of both dRobo1 and Comm leads to re-localization of dRobo1 from the cell surface to the ER/Golgi. **C**. Quantification of results for re-localization experiments. Cells transfected with the genes indicated were stained for dRobo1 or rRobo1. Subcellular localization at either the plasma membrane or predominantly in the ER/Golgi were scored by an experimenter blind to the plasmids present. At least 22 healthy cells as judged by nuclear staining were scored for each category. The percentage of cells with ER/Golgi localization is shown in the bar graph. Error bars are the 95% confidence interval to reflect sampling noise. Statistical significance relative to dRobo1 and rRobo1 controls is shown (*** p < 0.01, highly statistically significant) and was calculated using the Fisher exact test with two tails. For the PRRG and rRobo1, the Bonferroni correction was applied. Comparison of dRobo1 with and without Comm has a p value < 0.0001. Comparison of rRobo1 and PRRG4 has p < 0.0001. The PRRG3 and rRobo1 data are trending towards statistical significance, p = 0.0538 (cutoff value is p < 0.0125). **D**. rRobo1 in the presence of PRRG1 is localized predominantly to the cell surface. **E**. Co-expression of PRRG2 and rRobo1 results in cell surface localization of rRobo1. **F**. PRRG3 expression can result in a reduction in the level of rRobo1 on the cell surface. **G**. The majority of cells co-expressing PRRG4 and rRobo1 display an ER/Golgi localization for rRobo1. The underlying data are shown in S2 Data.

To verify the co-localization results, we chose to test the PRRG proteins' ability to clear rRobo1 from the cell surface in a blinded experiment in which COS cells were co-transfected with both genes of interest but the experimenter responsible for scoring only observed the dRobo1/rRobo1 staining. Comm and dRobo1 served as a positive control and Comm re-localized dRobo1 from the cell surface with 100% efficiency when scored blind (Fig 6A–6C; S2 Data). When *rRobo1* was co-expressed with the PRRG genes, PRRG4 prevented cell surface localization of rRobo1 or showed increased rRobo1 localization in the ER/Golgi over 80% of the time (p < 0.0001, two tailed Fisher’s exact test; Fig 6G; S2 Data). None of the other PRRG genes had a statistically significant effect on rRobo1 localization, although PRRG3 trended towards statistical significance, (p = 0.0538, cutoff value is p < 0.0125, Fig 6F), consistent with the mixed results obtained in the co-localization assay.

**Fig 7:**
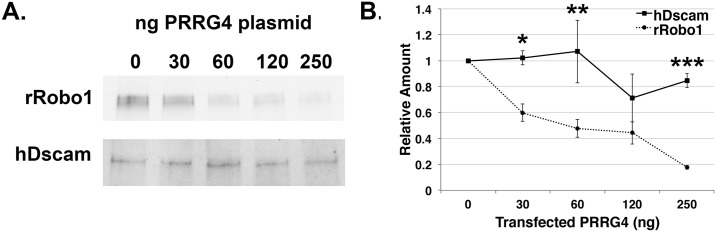
PRRG4 lowers rRobo1 protein levels in COS cells. COS cells were transfected with plasmids encoding either rRobo1 or hDscam and increasing amounts of PRRG4. **A**. Immunoblot analysis of hDscam and rRobo1 proteins levels in the presence of different amounts of PRRG4. Rat Robo1 protein levels fall with increasing amounts of PRRG4 plasmid, whereas hDscam levels remain relatively constant. **B**. Quantification of independent immunoblot experiments. The density of protein bands for hDscam and rRobo1 was quantified using ImageJ. The positive control was each plasmid transfected in the absence of PRRG4 plasmid and this densitometric value was set at one. All other values for each experiment were expressed as value relative to the control to normalize across different experiments. The values were analyzed in a one-way ANOVA with a Fisher LSD test. Asterisks represent p values for differences between hDscam and rRobo1, * p < 0.05, ** p < 0.01, *** p < 0.001. For 120ng of PRRG4, the difference was not statistically significant (p = 0.05118) but was clearly trending towards significance. The effect for 250ng PRRG4 was very strong and reproducible (p = 0.00002). Error bars represent standard error and are obscured by the 250ng PRRG4 data point for the dashed rRobo1 line. The underlying data are shown in S3 Data.

A dosage sensitive relationship between Comm and Robo has previously been demonstrated in cell culture, with increasing amounts of Robo plasmid leading to less Comm protein detectable by immunoblot [62]. We modified this assay to verify the PRRG4 result and found that increasing amounts of PRRG4 expression reduced rRobo1 levels as detected by immunoblot (Fig 7; S3 Data). We used the related immunoglobulin family member hDscam as a control and found negligible downregulation in the presence of PRRG4. 250ng of PRRG4 plasmid per well (9.5cm^2^) produced a very reliable down-regulation of rRobo1 compared to hDscam (p = 0.00002, one-way ANOVA, Fisher LSD test). Together these results indicate that PRRG4 downregulates Robo in COS-7 cells in a manner analogous to Comm.

## Discussion

The PRRG4 gene has been implicated in the autistic features of WAGR syndrome. Our work suggests that PRRG4 is a functional homologue of the *Drosophila commissureless* gene and may regulate the cell surface localization of the Robo guidance receptors and other molecules during human brain development.

How could haploinsufficiency for PRRG4 lead to autistic symptoms? The simplest explanation is that reduction in PRRG4 levels alters connectivity patterns in the developing brain due to increased Robo levels. Connectivity defects have been suggested as potentially underlying some cases of autism [63, 64]. Robos have been implicated in autism through single nucleotide polymorphism and expression studies [65–67]. It has been proposed that Robo gene variants are interfering with the serotonergic system, the anterior cingulate cortex or through a general effect on neurodevelopment. Additionally, alterations to the corpus callosum have also been implicated in autistic symptoms [68], and Robo/Slit signaling is required for corpus callosum formation [69, 70]. Robo /Slit signaling has been implicated in all aspects of neural development, not just axon guidance [71], so it is unclear at what stage of development PRRG4 function might be required. There is little information on the expression pattern of PRRG4 in the embryo, with the exception of Xenopus embryos in which expression appears quite broad and likely to include the CNS [72]. PRRG4 expression has been observed in Purkinje cells in the human cerebellum [19], neurons known to be important in autism models [73]. Embryonic *comm* expression is highly dynamic in the fly [1, 8], so thorough surveys of PRRG4 expression will be required to identify candidate regions for further analysis. In parallel, the development of knockout mice may also help identify affected brain areas. Identification of a PRRG gene that is expressed in spinal cord commissural neurons during axon crossing of the CNS midline would also establish whether the most well-known function of *comm* is conserved.

Comm is also required for the formation of *Drosophila* neuromuscular synapses, and is proposed to clear molecules from the cell surface to allow synaptogenesis to take place [11, 74, 75]. As autism appears to primarily be a synaptic disorder [76], haploinsufficiency for PRRG4 may disrupt synapse formation in WAGR syndrome. The synaptic function of Comm in flies has not been linked to regulation of Robo and likely involves unidentified molecules. The ubiquitin ligase Nedd4 is important for the synaptogenesis function and PRRG4 also binds Nedd4 proteins [18]. Additional proteins that interact with the PY motifs of PRRG4 have been observed, including the MAGI proteins, which are required for learning and memory [18]. PRRG4 could function as an adaptor protein regulating molecules acting at the synapse.

Our findings suggest that Comm may be γ-carboxylated and that γ-carboxylation could have arisen as a nervous system post-translational modification that was later co-opted for blood clotting. Surprisingly, an absence of γ-carboxylation leads to no phenotypic defects in the fly (Fig 4E) [44], and we have observed no effects of warfarin on embryonic development. If Comm is γ-carboxylated, then this modification is not required for embryonic function. In commissural neurons, Comm sorts Robo into vesicles destined for late endosomes and the lysosome [10]. Sorting may not require γ-carboxylation of Comm, or alternatively the putative Gla domain may have additional functions. We favor a model in which the cell surface localization of Comm/PRRG proteins will require γ-carboxylation and whereas trafficking from trans Golgi network to the lysosome will not.

Comm and PRRG proteins have been studied independently up to this point. The existence of molecular and genetic datasets for both genes will aid future experiments into the functions of these protein families. For example, the LPSY motif that binds Nedd4 is also required for Comm function in midline crossing. Additional binding partners for the LPSY motif have been identified [18], and these can be tested for functions in the fly CNS. Similarly, studies of *comm* in *Drosophila* and other species can guide expectations of PRRG4 function in WAGR syndrome [77, 78]. We were surprised to find that PRRG3 did not interact with rRobo1 at a statistically significant level in the cell clearance assay as we observed partial co-localization (Fig 5E). PRRG3 may be able to regulate Robo proteins in the exocytosis pathway, but less efficiently in endocytosis and will deserve further investigation. Interestingly, PRRG3 and PRRG4 both share a conserved cysteine in the transmembrane domain with insect Comm homologues (Fig 2C, highlighted in red and blue), whereas PRRG1 and PRRG2 do not. Our results suggest that Comm/PRRG proteins are part of an ancient family of cell surface protein regulators that originated in single celled eukaryotes and that a subset of WAGR syndrome symptoms are likely due to increased levels of cell surface proteins in axons or synapses.

## Materials and methods

### Drosophila stocks

The coding sequences of candidate genes *Rcr1* (NM_001178353), *Rcr2* (NM_001180311), *C17G10*.*7* (NM_062689), *CYYR1* (AF442733), *xShisa4* (NM_001096205), *PRRG1* (NM_027322), *PRRG2* (NM_022999), *PRRG3* (BC137616) and *PRRG4* (NM_178695) were synthesized with codon optimization for expression in *Drosophila* by Genscript. A C-terminal Myc epitope tag was added to each sequence and genes were delivered in pUC-57 with 5’ and 3’ restriction sites added to facilitate cloning into pUAST. Rcr1, Rcr2, CYYR1, xShisa4 and C17G10.7 coding sequences were subcloned into pUAST with EcoRI and XbaI. The *PRRG1-4* coding sequences were inserted as EcoRI-KpnI fragments. *Drosophila* injections were performed by Genetic Services Inc. or Rainbow Transgenics and transformants were selected and insertions mapped using standard methods. For construction of UAS-hRobo1, the human Robo1 clone described in Kidd et al. 1998 (Genbank #AF040990) was modified by PCR to change the stop codon to leucine (TGA to TTA), thereby introducing a HindIII site at the carboxy terminus of the protein. The original intention to insert an epitope tag appears to have failed. The hRobo1 gene was subcloned into the pUAST vector as an XbaI-HindIII fragment and used to transform *Drosophila* by standard techniques. *scabrous*-Gal4 and *scratch*-Gal4 were obtained from the Bloomington *Drosophila* Stock Center.

### Drosophila embryos and immunostaining

Drosophila embryos were processed and immunostained as previously described [79]. The following antibodies were used: mouse anti-c-Myc 9E10 (Santa Cruz) 1:200, BP102 (DSHB) 1:10, mouse monoclonal antibody 13C9 against fly Robo1 (DSHB) 1:20, rabbit anti-hRobo1 (Abcam ab7279) 1:1000. Anti-mouse (1:500) and rabbit (1:1000) HRP- conjugated secondary antibodies were obtained from Jackson Laboratories. For phenotypic comparisons, transgene presence was confirmed by immunostaining.

### Cell culture and western blotting

*C-terminal myc-tagged Rcr1*, *Rcr2*, *PRRG1*, *PRRG2*, *PRRG3* and *PRRG4* synthetic sequences were subcloned into pcDNA3.1 (Life Technologies) for expression in mammalian cells. *Drosophila Robo1* in pcDNA is described in [80]. HA-tagged rat *Robo1* in pCS2+ was a gift from Yi Rao (National Institute of Biological Sciences, Beijing University) to Grant Mastick (University of Nevada, Reno). *GFP-Comm* in pcDNA was provided by Daniela Rotin (Peter Gilgan Centre for Research and Learning, Toronto). Myc-tagged human *Dscam* in pcDNA was a gift from K.-L. Guan (Pharmacology, UCSD). COS-7 cells were transfected using Lipofectamine 3000 (Life Technologies) and analyzed 48 hours post-transfection for all cell culture experiments. To assay re-localization of Robo in response to Comm/PRRG proteins, 500 ng of Robo plasmid alone or with 250ng candidate gene plasmid were added to each well of a six well plate. For immunocytochemistry, cells were washed with PBS then fixed in 4% PFA. Cells were blocked in 5% NGS for 30 minutes prior to antibody labeling. Antibodies used for immunocytochemistry were mouse anti-c-Myc 9E10 (Santa Cruz) 1:200, rabbit anti-HA (Covance) 1:250, mouse monoclonal antibody 13C9 against fly Robo1 (DSHB) 1:20. Secondary detection used Alexa Fluor anti-rabbit 488 and anti-mouse 568 (Jackson Laboratories). To assay total levels of rRobo1 and hDscam protein in the presence of PRRG4, 500ng of rRobo1 or hDscam plasmid alone and with increasing amounts of PRRG4 plasmid were transfected per well of six well plates, as described in [62]. After 48 hours cells were harvested and lysed in ice cold lysis buffer containing 50mM HEPES (pH 7.2), 100mM NaCl, 1mM MgCl2, 1mM CaCl2 and 1% NP-40 with protease inhibitors [81]. Total protein content was normalized using a BCA Protein Assay Kit (Thermoscientific, Pierce). Protein was separated on a 4–20% gradient SDS-PAGE gel and electroblotted to nitrocellulose membrane (Bio-Rad). Membranes were blocked in 5% milk with 0.1% Tween 20 and subsequently incubated with monoclonal antibody 13C9 (DSHB, 1:20), rabbit anti-HA (Covance, 1:1000) or anti-C-Myc (Santa Cruz, 9E10 1:250) (to confirm increasing levels of PRRG4 protein). Proteins were detected using HRP-conjugated secondary antibodies (Jackson Laboratories, 1:5000) and visualized with ECL detection reagents in a ChemiDoc imager (Bio-Rad). Signal intensities were measured in ImageJ.

## Supporting information

S1 Fig Candidate *Drosophila* Shisa-like protein: CG15760. The majority of Shisa-like proteins are characterized by six Cys amino acids in a C*C*CC*CC pattern [40]. CG15760 consists of six Cys residues in this arrangement immediately after a putative signal sequence. The transmembrane domain has a four aromatic amino acid motif found in Shisa-like proteins. Combined with PY motifs, these features place CG15760 into the Shisa-like family of proteins, which also includes the WBP1, VOPP1 and TMEM92 protein families.
(PDF)

S2 Fig Alignment of *commissureless* and *C*. *elegans C17G10*.*7*. C17G10.7 is a putative *C*. *elegans* protein that displays similarity to Comm in the putative propeptide ITFEI motif, transmembrane domain, LPSYDEAL motif and conservation at the carboxy terminal. An alternative interpretation is that C17G10.7 is a four transmembrane protein resembling vertebrate LAPTM4 proteins [39].
(PDF)

S3 Fig Co-localization of PRRG3 and rRobo1 in COS cells. COS cells were co-transfected with plasmids for PRRG3 and rRobo1 and stained with fluorescent immunohistochemistry. Cell nuclei were stained with DAPI (blue), PRRG3 is red (anti-myc epitope tag) and rRobo1 is green (anti-HA epitope tag). In most examples, rRobo1 is localized to the cell surface, but several cases staining is predominantly in the ER/Golgi.
(TIF)

S1 Data Quantification of CNS axon guidance defects. Embryonic nerve cords stained with BP102 were analyzed using the criteria in Table 1. The number of segments displaying a particular phenotype in each embryo analyzed are recorded in this table.
(XLSX)

S2 Data Cellular localization of Robo proteins in COS cells. COS cells transfected with plasmids encoding fly *robo1*, *rRobo1*, *comm* and *PRRG1-4* were immunostained for Robo expression. Healthy cells for each genotype were scored by an experimenter blind to the genotype of the cell. The scoring categories were PM for plasma membrane localization or ER for endoplasmic reticulum/Golgi localization or PM/ER when both localizations were present.
(XLSX)

S3 Data Densitometric analysis of immunoblots. Images of protein bands (rRobo1, hDscam) in the presence of different amounts of PRRG4 were quantified in ImageJ. For each experiment the value for the band in which no PRRG4 was co-transfected was set to an arbitrary value of 1. All other values were expressed as values relative to 1 and are recorded in this table.
(XLSX)
