# Correction: The WAGR syndrome gene PRRG4 is a functional homologue of the *commissureless* axon guidance gene

**DOI:** 10.1371/journal.pgen.1007061

**Published:** 2017-10-23

**Authors:** Elizabeth D. Justice, Sarah J. Barnum, Thomas Kidd

The following information is missing from the funding section: This study is also funded by the National Institutes of Health grant P20 GM103554 (https://www.nigms.nih.gov).
