# Supplementary material for: The WAGR syndrome gene PRRG4 is a functional homologue of the commissureless axon guidance gene
Source: PLoS Genet. 2017 Aug 31;13(8):e1006865. doi: 10.1371/journal.pgen.1006865 (PMC5578492; doi:10.1371/journal.pgen.1006865)
Supplement: S1 Fig — The majority of Shisa-like proteins are characterized by six Cys amino acids in a C*C*CC*CC pattern [40]. CG15760 consists of six Cys residues in this arrangement immediately after a putative signal sequence. The transmembrane domain has a four aromatic amino acid motif found in Shisa-like proteins. Combined with PY motifs, these features place CG15760 into the Shisa-like family of proteins, which also includes the WBP1, VOPP1 and TMEM92 protein families. (PDF) [file pgen.1006865.s001.pdf]

***Drosophila melanogaster* CG15760 is a candidate invertebrate Shisa-like protein**

MWLRPSPSHTNALQCLLLSLFLLLGLLPANVSARFCEGCTQGCCTQGCCPPYQGGPRPLPPSSDSHVLNL
FFATHWFFWCVVVAIILAILCAYSLWKKRRTLCGWGFNEHHTQSEGDSAGSCYAPPQYSRCNSFHHPPPP
YTEVTSKPDLYPLVFTCNSDSGNGKGNGSSSYLMSQSDQHFRYMANSSSSLSDIFVNNSCVAGMLPESGG
AGGAFHGAESGSATQAASLNSLAMGGLGGSVTGAAGTSGGSGTPVIYSNGCIQPNPLHSLENCCQPSAQV
SGVSSLANIGTPGSPPQATSPTGEVREILDQIRQLQAGVSYEQLLLNGTGAKPRLQHTLSTPSNSQQVLQ
PILTTSASISGGTSGRSKKFFTSGSKTQPNKALYIPMAAIGGSGSGAQRCVLKSPVSSVVSGASFFVNRG
RVARKGWITRSAPTTPGSALPPNRLGDDSPLLLNEHDEDAIDDDEDEEQEVVDRQRNHQETQRGHEDTDS
ETEAEARNHRRQHRHHRGHRGHRRGQLE

CEGCTQGCCTQGCC: Six cysteine motif characteristic of Shisa-like proteins
WFFW: motif consisting of four aromatic residues found in Shisa-like proteins
PPPY: PY motifs
Putative signal sequence
Transmembrane domain
