# Supplementary material for: The WAGR syndrome gene PRRG4 is a functional homologue of the commissureless axon guidance gene
Source: PLoS Genet. 2017 Aug 31;13(8):e1006865. doi: 10.1371/journal.pgen.1006865 (PMC5578492; doi:10.1371/journal.pgen.1006865)
Supplement: S2 Fig — C17G10.7 is a putative C. elegans protein that displays similarity to Comm in the putative propeptide ITFEI motif, transmembrane domain, LPSYDEAL motif and conservation at the carboxy terminal. An alternative interpretation is that C17G10.7 is a four transmembrane protein resembling vertebrate LAPTM4 proteins [39]. (PDF) [file pgen.1006865.s002.pdf]

Alignment of Commissureless and C. elegans C17G10.7

```
Comm   MISTTDYPTVETTTTAEELYAEYISAPASSMSPAAIAEHLQQNQITFEIPSAHDLRHIDALNSFNALLQRIGNAAVSYDPAPP
C17G10    MDHSTRFRVHNWLRSRRKLFQSRMCLLM-LVMI-NFVNSVILTLEICGE-DSNMTLLM-FFVNTF---VVFLSMYGL---
                             + S     M    I        +T+EI    D         F             Y

Comm   SGWSPDGSISTEQLSKSVVLDLADLRDRSEESGESSWWSQIFGDADMHVIINYLWIGVVSSLVILSLVFILFSCYFYR-KFRTWKK
C17G10 ------------YNFRPVFLSPNVLLKIILSSSAL-FYGLQMAETTSNSAIFV-WLTISIVFFILEIHTM-FSTTFDIIKQLNLR-
                      + V+L    L      S    ++   +       ++ + W+ +   ++IL + ++ FS++F++ K----+

Comm    CNKDIRAQIHAASDSYSSHLVGCDASRLLLHQQMQHPHHRSSEAGFYQIESPPCYT----IATGLPSYDEALHH--QPR
C17G10 AYSK-----------------------------------------------PPPNYNQVMSIDVPPPSYEEALVRLEQNR
          K                                                PP Y     I    PSY+EAL    Q R

Comm   HFAYGMKFVYPSLAAVHHHHHCISNWEKQEPLNKLQKCKLSAAAAVEEDKADSSSSTSASASPSSSESSNLA
C17G10 ---TAAKNQSS-------------------------------------------------------------
             K

Comm   TATPAICINMPSGRQDEEVDNSDSDSAIAVAVAVAQSLQPAAPADDDCASLVVVVAA
C17G10 ----------------------------------PKPDQPAQLNTCDLAHLVV
                                             QPA     D   LVV
```

Alternative interpretation of C17G10.7 as a four-pass transmembrane protein
Transmembrane domains are highlighted in blue.

```
C17G10.7   MDHSTRFRVHNWLRSRRKLFQSRMCLLMLVMINFVNSVILTLEICGEDSNMTLLMFFVNTFVVFLSMYGL

C17G10.7   YNFRPVFLSPNVLLKIILSSSALFYGLQMAETTSNSAIFVWLTISIVFFILEIHTMFSTTFDIIKQLNLR

C17G10.7   AYSKPPPNYNQVMSIDVPPPSYEEALVRLEQNRTAAKNQSSPKPDQPAQLNTCDLAHLVV
```
