# Supplementary figures and images for: The WAGR syndrome gene PRRG4 is a functional homologue of the commissureless axon guidance gene
Source: PLoS Genet. 2017 Aug 31;13(8):e1006865. doi: 10.1371/journal.pgen.1006865 (PMC5578492; doi:10.1371/journal.pgen.1006865)

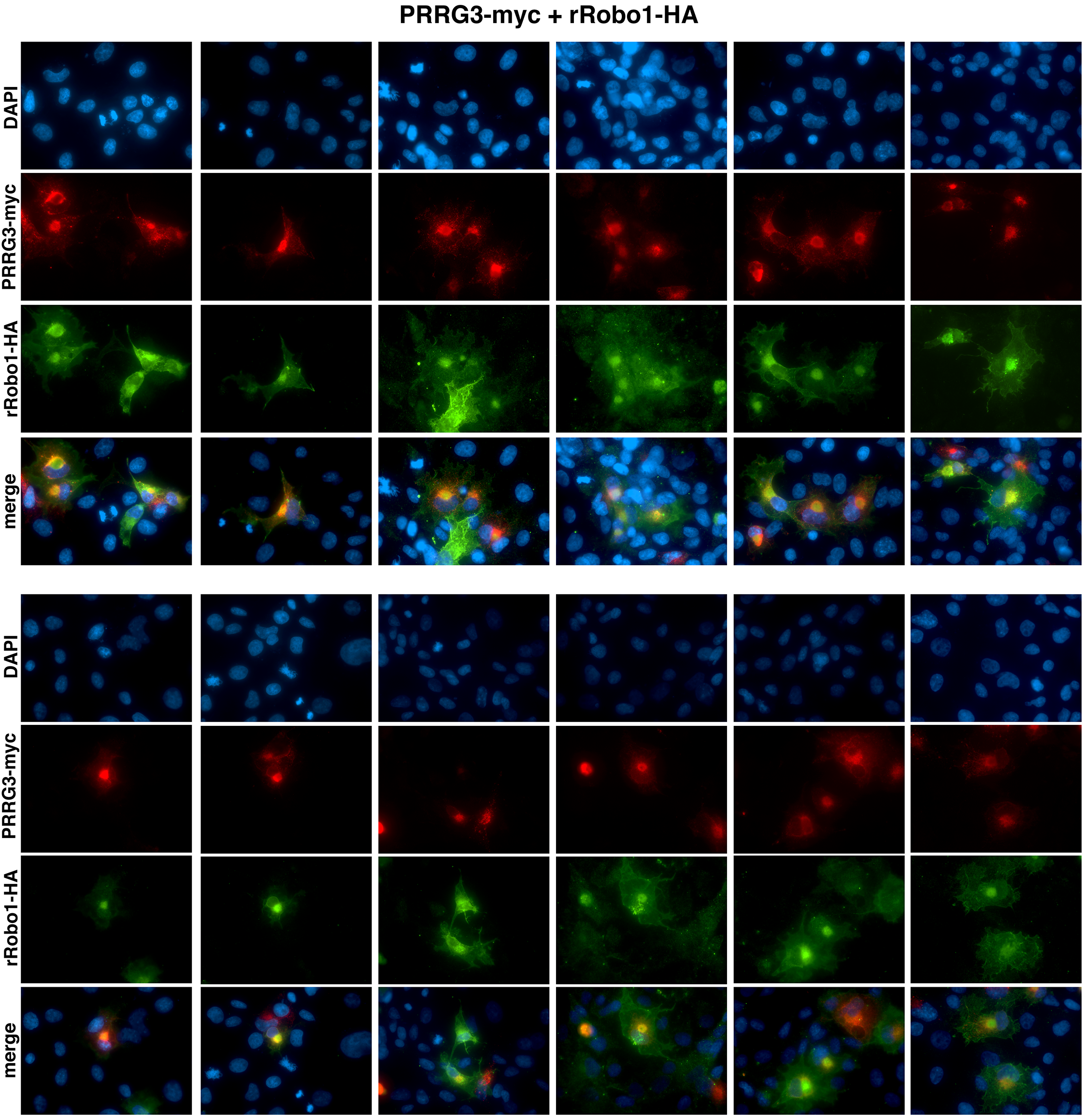

Supplement: S3 Fig — COS cells were co-transfected with plasmids for PRRG3 and rRobo1 and stained with fluorescent immunohistochemistry. Cell nuclei were stained with DAPI (blue), PRRG3 is red (anti-myc epitope tag) and rRobo1 is green (anti-HA epitope tag). In most examples, rRobo1 is localized to the cell surface, but several cases staining is predominantly in the ER/Golgi. (TIF) [file pgen.1006865.s003.tif]
